# Four-year follow-up of psychiatric and psychosomatic profile in patients with Inflammatory Bowel Disease (IBD)

**DOI:** 10.1186/s40359-024-01726-5

**Published:** 2024-04-17

**Authors:** Sara Gostoli, Francesco Ferrara, Ludovica Quintavalle, Sara Tommasino, Graziano Gigante, Maria Montecchiarini, Alessia Urgese, Francesco Guolo, Regina Subach, Angelica D’Oronzo, Annamaria Polifemo, Federica Buonfiglioli, Vincenzo Cennamo, Chiara Rafanelli

**Affiliations:** 1 Department of Psychology “Renzo Canestrari”, University of Bologna, Bologna, Italy; 2 Gastroenterology and Interventional Endoscopy Unit, AUSL Bologna, Bologna, Italy; 3 Division of Cardiology, Bellaria Hospital, AUSL Bologna, Bologna, Italy

**Keywords:** Diagnostic Criteria for Psychosomatic Research (DCPR), Inflammatory Bowel Disease (IBD), Lifestyle behaviors, Psychological well-being, Psychiatric diagnoses, Psychosomatic syndromes

## Abstract

Psychological characterization of patients affected by Inflammatory Bowel Disease (IBD) focuses on comorbidity with psychiatric disorders, somatization or alexithymia. Whereas IBD patients had higher risk of stable anxiety and depression for many years after the diagnosis of the disease, there is a lack of studies reporting a comprehensive psychosomatic assessment addressing factors of disease vulnerability, also in the long-term. The objective of this investigation is to fill this gap in the current literature. The aims were thus to assess: a) changes between baseline and a 4-year follow-up in psychiatric diagnoses (SCID), psychosomatic syndromes (DCPR), psychological well-being (PWB-I), lifestyle, gastrointestinal symptoms related to IBD and Irritable Bowel Syndrome (IBS)-like symptoms b) stability of psychiatric and psychosomatic syndromes at 4-year follow-up. A total of 111 IBD outpatients were enrolled; 59.5% of them participated at the follow-up. A comprehensive assessment, including both interviews and self-report questionnaires, was provided at baseline and follow-up. Results showed increased psychiatric diagnoses, physical activity, consumption of vegetables and IBS-like symptoms at follow-up. Additionally, whereas psychiatric diagnoses were no longer present and new psychopathological pictures ensued at follow-up, more than half of the sample maintained psychosomatic syndromes (particularly allostatic overload, type A behavior, demoralization) from baseline to follow-up. Long-term presence/persistence of such psychosocial burden indicates the need for integrating a comprehensive psychosomatic evaluation beyond traditional psychiatric nosography in IBD patients. Moreover, since psychosomatic syndromes represent vulnerability factors of diseases, further studies should target subgroups of patients presenting with persistent psychosomatic syndromes and worse course of the disease.

## Introduction

Inflammatory Bowel Diseases (IBD), including ulcerative colitis (UC) and Crohn's disease (CD), are chronic diseases that exhibit an unpredictable clinical course, with alternating active phases and periods of variable duration of remission [1, 2]. The epidemiology of IBD is rapidly changing worldwide. The estimated prevalence continues to rise in Western countries, with a high burden of IBD in North America, Oceania, and Europe [3], reaching approximately 2.2 million of IBD patients in USA [4]. In Northern Italy, the average incidence of IBD registered in the period 2016–2021 was 18 per 100.000 inhabitants/year [5]. IBD specific symptoms are fever, weight and appetite loss, bloody stool, vomiting and anemia [6, 7]. A high percentage of other symptoms such as abdominal pain, bloating, diarrhea and watery stools, transversally exhibited in IBD and Irritable Bowel Syndrome (IBS) patients are reported. IBD may be complicated by extra-intestinal systemic manifestations such as rheumatological, ocular, dermatological, hepatobiliary, pulmonary, cardiac, and hematological [8]. There is also an increased risk of neoplasms, mainly colorectal [9]. An intimate connection between the central nervous and the enteric nervous systems (gut-brain axis) was observed [10]. Indeed, psychiatric comorbidity in IBD is well known and higher than in matched cohorts [11, 12]. It is increasingly acknowledged that both psychological and biological pathways play a role in stress-induced alterations in IBD expression [13]. On the one hand, psychiatric disorders such as anxiety and depressive disorders may trigger and exacerbate intestinal inflammation or contribute to relapse [14, 15]. On the other hand, both active physical symptoms and the chronic and severe nature of IBD can negatively impact an individual's mental health [16, 17]. Furthermore, subclinical manifestations, such as somatization [18] and alexithymia [19], affecting IBD patients’ clinical disease activity [18] and quality of life [20, 21] were observed. In IBD patients, healthy lifestyle such as regular physical exercise [22], a personalized dietary approach, [23], avoidance of smoking [24, 25] and light or no alcohol consumption [26] are recommended. However, some of these recommendations are contrary to what is often practiced by IBD patients [27]. Whereas Bisgaard and colleagues [28] found that patients with IBD had higher risk of anxiety and depression starting at least five years before and continuing until at least ten years after IBD diagnosis, there is a lack of studies reporting a comprehensive psychosomatic assessment, addressing different factors of disease vulnerability in IBD patients, also in the long-term, despite the advocated need for a thorough evaluation and management of IBD [28]. In order to fill the gaps in the existing literature, the present study aimed to conduct a more extensive investigation of the clinical and subclinical psychological profile of IBD outpatients at baseline and at a 4-year follow-up. Additionally, our study aimed to verify the long-term stability of psychosomatic syndromes in IBD patients. Specifically, the present study had two objectives: a) to assess changes between baseline and a 4-year follow-up in psychiatric profile according to DSM-5 [29], psychosomatic syndromes on the basis of Diagnostic Criteria for Psychosomatic Research (DCPR) [30], psychological well-being (PWB) according to PWB-Interview (PWB-I) [31] and lifestyle, as well as in IBD-related symptoms and IBS-like symptoms b) to assess the stability of psychiatric and psychosomatic syndromes at 4-year follow-up.

## Methods

### Participants and procedure

Consecutive outpatients with IBD in remission phase, including Crohn’s disease and ulcerative colitis, were recruited at the Unit of Gastroenterology of Local Unit Health (AUSL) of Bologna (Italy), after a check-up visit. The diagnosis of CD and UC were based on clinical history, blood and fecal tests, endoscopic, histologic and radiologic findings, following the ECCO-ESGAR Guidelines [32, 33].

Patients were recruited at baseline between December 2018 and January 2020. After the check-up visit, patients were asked to join the study and the informed consent document was provided. The interview was conducted soon after obtaining patients’ written informed consent. Participants were subsequently contacted again, approximately four years later, to set an appointment for the follow-up interview, which was conducted by phone. The local ethics committee approved the longitudinal study. All procedures involved in the present investigation adhere to the ethical standards set by the relevant national and institutional committees on human experimentation and with the Helsinki Declaration of 1975, as revised in 2008.

### Assessment

The interviews followed the same procedure at baseline and follow-up. Patients were assessed by three clinical psychologists trained to conduct the interviews. During the baseline assessment, patients underwent face-to-face interviews, whereas, during the follow-up, interviews were conducted via telephone. At both assessments, sociodemographic data, including gender, age, education level, occupation, marital status were collected, as well as regularly taken medications.

Participants underwent three validated clinical interviews for the assessment of psychiatric disorders, psychosomatic syndromes and psychological well-being. Information about lifestyle such as physical activity, diet, alcohol consumption, and smoking were collected as well. Additional ad hoc questions about the presence of IBD related symptoms and IBS-like symptoms were introduced. On average, the interview lasted 45 minutes.

#### Psychiatric diagnoses

The Italian adaptation [34] of the Structured Clinical Interview for DSM-5, clinician version (SCID-5-CV) [29, 35], to assess the presence of various psychiatric conditions, including depressive disorders (major depression), anxiety disorders (panic disorder, generalized anxiety disorder, agoraphobia and social anxiety), eating disorders (bulimia, binge eating disorder, and anorexia nervosa), and other disorders such as obsessive-compulsive disorder, somatic symptom disorders and illness anxiety, was employed. Moreover, current minor depression from the DSM-IV-TR appendix was assessed [36]. The SCID-5-CV demonstrated exceptional reliability, notable specificity, and clinical validity [37]. These findings substantiate its utility and appropriateness for routine clinical application [38].

#### Psychosomatic syndromes

The Italian version of the Semi-Structured Interview, which is based on the revised Diagnostic Criteria for Psychosomatic Research (DCPR-R) [30], was employed for the identification of psychosomatic syndromes. The updated version of DCPR was developed based on insights derived from its extensive use in many patients and various clinical settings [39, 40], encompassing diagnostic criteria for two additional syndromes such as allostatic overload and hypochondriasis. The interview protocol, grounded in DCPR-R, has already demonstrated its utility within medical settings [41]. It comprises a set of dichotomous (yes or no) items, complemented with screening questions that refer to the essential criteria of the disorders. This assessment instrument enables the examination of psychosomatic syndromes, which are delineated into four distinct domains: stress, personality, illness behavior, and psychological manifestations [30]. The stress domain includes allostatic overload. In the personality domain, type A behavior and alexithymia are considered. Illness behavior encompasses hypochondriasis, disease phobia, thanatophobia, health anxiety, persistent somatization, conversion symptoms, anniversary reaction and illness denial. The cluster regarding the psychological manifestations covers demoralization, demoralization with hopelessness, irritable mood and somatic symptoms secondary to a psychiatric disorder [30]. These criteria have been meticulously developed with the aim of operationalizing the spectrum of manifestations of illness behavior and sub-threshold distress within both psychiatric and medical contexts. They can be utilized independently of, or in conjunction with, the DSM criteria [42]. The interview has demonstrated excellent interrater reliability, construct validity, and predictive validity concerning psychosocial functioning and treatment outcomes [43, 44].

#### Psychological well-being

The Italian version (PWB-I) [31] of the semi-structured interview based on the Psychological Well-Being (PWB) scales items [45] was employed. This instrument was utilized to evaluate the psychological well-being and resilience of individuals in clinical populations, aligning with the multidimensional model proposed by Ryff [45]. The original version of the PWB scale has exhibited favorable psychometric properties, including robust internal consistency [46] and this adaptation shows test-retest reliability in prior research endeavors [31]. It was used to facilitate tailoring of interventions and support strategies to address specific psychological needs of patients affected by IBD. The adapted questionnaire consists of 18 questions, in which respondents are asked to provide dichotomous responses in the form of "Yes" or "No" to each of these questions that encompass the six fundamental dimensions of PWB (environmental mastery, personal growth, purpose in life, autonomy, self-acceptance and positive relationship with others) as conceptualized by Ryff [45].

#### Lifestyle-related behaviors

An adapted version of the GOSPEL questionnaire to assess lifestyle-related behaviors [47, 48] was used. This particular adaptation was specifically crafted to surmount the inherent limitations posed by comprehensive questionnaires typically employed for the evaluation of dietary habits and leisure time physical activity. The assessment instrument is multifaceted and includes lifestyle dimensions such as physical activity, dietary habits (vegetables, fruits, fish and dairy products consumption), alcohol consumption and tobacco smoking. Respondents were asked to rate their habits on a 4-point Likert scale, indicating the frequency of each specific habit per week. This measure is frequently well-suited for real-world clinical environments marked by time limitations and practical constraints [47, 48]. This modified questionnaire has demonstrated its efficacy in previous investigations focused on patients affected by various medical conditions [49, 50].

#### IBD-related symptoms, IBS-like symptoms

In order to assess IBD-related symptoms (fecal blood) and IBS-like symptoms (e.g. constipation/diarrhea, mild abdominal pain, bloating) both at baseline and follow-up, five ad hoc questions were asked.

### Data analysis

Data were entered into SPSS 20.0 for Windows (SPSS Inc., Chicago, IL, USA). Descriptive analyses were used to outline socio-demographic data, pharmacological treatment, psychiatric/psychosomatic diagnoses (DSM-5 and DCPR-R), well-being scores (PWB-I), lifestyle-related behaviors, IBD symptoms and IBS-like symptoms of the sample both at baseline and follow-up. Normal distribution of data was assessed with Shapiro-Wilk test and, when not confirmed (*p* < .05), median and interquartile range was reported.

For both first and second aim, analyses were conducted on the subgroup of patients who underwent follow-up assessment.

Regarding the first aim, differences in the frequencies of current psychiatric diagnoses (DSM) and psychosomatic syndromes (DCPR) between baseline and follow-up were evaluated using the McNemar test. Wilcoxon signed-rank test was applied to underline differences between the number of DCPR syndromes and the number of SCID diagnoses at baseline and follow-up. Next, differences in well-being scores (PWB-I) and lifestyle-related behaviors from baseline to follow-up, were studied using the Sign test. Additionally, to assess differences in the distribution of smokers and non-smokers between baseline and follow-up, a McNemar test was employed. Finally, a McNemar test with the aim of comparing the presence of IBD related and IBS-like symptoms in the two evaluations was applied.

Regarding the second aim, to determine which DSM and DCPR diagnoses were maintained between baseline and follow-up, contingency tables were used. Significance level for all the analyses was set to 0.05, two-tailed.

## Results

At baseline, the sample consisted of 111 patients: 44 males (39.6%) and 67 females (60.4%). The distribution of age (*W* = .974, *p* = .029) and duration of the disease (*W* = .895, *p* < .001) did not approximate a normal distribution. Patients had a median age of 49 years (*IQR* = 22) and have been living with the disease for a median of 9 years (*IQR* = 12).

Out of the 111 patients contacted to be assessed in the follow-up study, 59.5% agreed to participate. 36.4% were males and 63.6% females. The distribution of age (*W* = .963, *p* = .044) and duration of the disease (*W* = .863, *p* <.001) did not approximate a normal distribution. Participants had a median age of 50 years (*IQR* = 23) and have been living with the disease for a median of 13 years (*IQR*= 12). Out of the 111 patients, 45 (40.5%) did not participate due to time constraints (33.4%) and inability to be contacted (6%); among them, 20 (44.4%) were males and 25 (55.6%) females with a mean age (W = .967, *p* =.217) of 55.3 years (*SD* = 13.20). Patients’ sociodemographic data and pharmacological treatment of baseline and follow-up are described in Table 1.

**Table 1:** Socio-demographic data and pharmacological treatment of the sample at baseline and follow-up (*N*= 66)

Sample characteristics	Baseline		Follow-up	
	*n*	*%*	*n*	*%*
Sex				
Male	24	36.4%	24	36.4%
Female	42	63.6%	42	63.6%
Disease				
Ulcerative colitis	41	62.1%	41	62.1%
Crohn’s disease	25	37.9%	25	37.9%
Marital status				
Single	23	34.8%	19	28.8%
Married	39	59.1%	43	65.2%
Divorced	4	6.1%	4	6.1%
Widowed	0	0	0	0
Employment status				
Retired	7	10.6%	13	19.7%
Manual labor	23	34.8%	15	22.7%
Sedentary work	30	45.5%	30	45.5%
Unemployed	6	9.1%	8	12.1%
Education				
Primary school certificate	0	0	0	0
8th grade diploma	21	31.8%	19	28.8%
Diploma	28	42.4%	30	45.5%
Bachelor’s degree	5	7.6%	3	4.5%
Master degree	10	15.2%	12	18.2%
PhD	2	3%	2	3%
Pharmacological Treatment				
Antidepressants	7	10.6%	4	6.1%
TCA	0	0	1	1.5%
SSRI	5	7.6%	2	3%
Other antidepressants	2	3%	1	1.5%
Antipsychotics	0	0	1	1.5%
Anxiolytics	5	7.6%	2	3%
Azathiaprina	4	6.1%	2	3%
Biological	3	4.5%	21	31.8%
Cortisone	7	10.6%	10	15.2%
Mesalazine	57	86.4%	44	66.7%
Anticoagulant	2	3%	4	6.1%
Antidyslipidemics	2	3%	7	10.6%
Antidiabetics	1	1.5%	0	0
Antihypertensive	8	12.1%	8	12.1%

*PhD* Philosophiae Doctor, *TCA* Tricyclic Antidepressant, *SSRI* Selective Serotonin Reuptake Inhibitors

Regarding the first aim of the study, the frequency of DSM diagnoses from baseline to follow-up showed a significant increase (*p* < .001). Indeed, 6.1% of IBD patients presented with DSM diagnoses at baseline, whereas 31.8% at follow-up (frequencies associated with each diagnosis, both at the baseline and follow-up assessments, are delineated in Table 2).

**Table 2:** Frequency of psychiatric and psychosomatic diagnoses at baseline and follow-up, compared using the McNemar test. Frequency of maintenance and development of diagnoses between baseline and follow-up is also outlined

	Baseline	Maintained	Developed	Follow-up	
Psychiatric diagnoses	*n (%)*	*n (%)*	*n (%)*	*n (%)*	*p*
Current major depression	2 (3.03%)	1 (1.5%)	4 (6.06%)	5 (7.6%)	.375
Current minor depression	0 (0%)	0 (0%)	10 (15.15%)	10 (15.15%)	.
Current panic disorder	1 (1.5%)	0 (0%)	2 (3.03%)	2 (3.03%)	1
Agoraphobia	0 (0%)	0 (0%)	3 (4.5%)	3 (4.5%)	.
Social Anxiety	0 (0%)	0 (0%)	5 (7.6%)	5 (7.6%)	.
Generalized anxiety disorder	0 (0%)	0 (0%)	8 (12.12%)	8 (12.12%)	.
Obsessive-Compulsive Disorder	0 (0%)	0 (0%)	4 (6.06%)	4 (6.06%)	.
Bulimia	0 (0%)	0 (0%)	0 (0%)	0 (0%)	.
Binge Eating Disorder	0 (0%)	0 (0%)	2 (3.03%)	2 (3.03%)	.
Anorexia	0 (0%)	0 (0%)	1 (1.5%)	1 (1.5%)	.
Somatic Symptoms disorder	1 (1.5%)	0 (0%)	5 (7.6%)	5 (7.6%)	.219
Illness Anxiety	1 (1.5%)	0 (0%)	5 (7.6%)	5 (7.6%)	.219
Psychosomatic syndromes	*n (%)*	*n (%)*	*n (%)*	*n (%)*	*p*
Allostatic overload	18 (27.3%)	13 (19.7%)	12 (18.2%)	25 (37.9%)	.143
Alexithymia	17 (25.7%)	5 (7.6%)	19 (28.8%)	24 (36.3%)	.281
Health Anxiety	3 (4.5%)	0 (0%)	1 (1.5%)	1 (1.5%)	.625
Type A behavior	19 (28.8%)	11 (16.7%)	6 (9.1%)	17 (25.7%)	.791
Disease phobia	2 (3.03%)	0 (0%)	0 (0%)	0 (0%)	.
Hypochondriasis	1 (1.5%)	0 (0%)	2 (3.03%)	2 (3.03%)	1
Thanatophobia	1 (1.5%)	0 (0%)	0 (0%)	0 (0%)	.
Illness denial	2 (3.03%)	0 (0%)	1 (1.5%)	1 (1.5%)	1
Persistent somatization	4 (6.06%)	1 (1.5%)	15 (22.7%)	16 (24.24%)	.008**
Demoralization	9 (13.6%)	5 (7.6%)	9 (13.6%)	14 (21.2%)	.267
Demoralization with hopelessness	2 (3.03%)	1 (1.5%)	4 (6.1%)	5 (7.6%)	.375
Irritable mood	8 (12.1%)	2 (3.03%)	4 (6.1%)	6 (9.1%)	.754
Conversion symptoms	0 (0%)	0 (0%)	5 (7.6%)	5 (7.6%)	.
Functional somatic symptoms secondary to a psychiatric disorder	1 (1.5%)	0 (0%)	0 (0%)	0 (0%)	.
Anniversary reaction	1 (1.5%)	0 (0%)	2 (3.03%)	2 (3.03%)	1

^*^p < .05. **p < .01

Regarding psychosomatic syndromes, the frequency of DCPR diagnoses between baseline and follow-up did not show a significant increase (*p =* .*086*). 71.2% of IBD patients presented at least one DCPR syndrome at baseline, whereas 75.5% at follow-up. The only significant change was the increase in persistent somatization: 6.06% at baseline and 24.24% at follow-up (*p* = .008) (Table 2).

No significant results emerged from the comparisons of PWB-I scores between baseline and follow-up (Table 3)*.*

**Table 3:** PWB-I median scores and relative interquartile ranges of participants at baseline compared with follow-up using Sign test

PWB-I Scores	Baseline		Follow-up		*p*
	*Mdn*	*IQR*	*Mdn*	*IQR*	
PWB-I environmental mastery	3	0	3	1	.664
PWB-I personal growth	2.5	1	2	1	1
PWB-I purpose in life	2	1	2	1	1
PWB-I autonomy	2	1	2	1	.241
PWB-I self-acceptance	3	0	3	1	.152
PWB-I positive relations	3	1	3	1	.860
PWB-I tot	15	3	14.5	4	.596

*PWB-I* Psychological Well-Being Interview

^*^p < .05. **p < .01

The analysis of lifestyle-related behaviors showed few significant differences between baseline and follow-up. In particular, the frequency of physical activity (*Z* = - 2.09, *p* = .037) and the consumption of vegetables (*Z* = - 2.96, *p* = .003) increased, while no significant changes between baseline and follow-up were observed for the remaining habits. Lifestyle data and comparisons are reported in Table 4*.*

**Table 4:** Comparison of lifestyle habits between baseline and follow-up, using Sign Test

Lifestyle habit	Median Frequency			
	Baseline	Follow-up	*Z*	*p*
Vegetables consumption	Two / Three times a week	Two / Three times a week	- 2.96	.003**
Fruit consumption	Everyday	Everyday	- 0.86	.391
Fish consumption	Two / Three times a week	Two / Three times a week	- 0.39	.700
Dairy products consumption	Two / Three times a week	Two / Three times a week	- 1.46	.144
Yogurt consumption	Never / Occasionally	Never / Occasionally	0	1
Alcohol consumption	Never / Occasionally	Never / Occasionally	- 0.17	.864
Physical activity	Never / Occasionally	Once / Twice a week	- 2.09	.037*

^*^p < .05. **p < .01

Concerning IBD related symptoms, results did not show any significant change. 7.6% presented IBD related symptoms at baseline, whereas 16.7% at follow-up (no one maintained these symptoms). About IBS-like symptoms, patients showed a significant increase between baseline and follow-up (*p* < .001). Indeed, 9.1% at baseline presented IBS-like symptoms, whereas 56.1% at follow-up (4.5% maintained the symptoms) (Table 5).

**Table 5:** IBD-related symptoms and IBS-like symptoms at baseline and follow-up, compared using the McNemar test. Frequency of maintenance and development of symptoms between baseline and follow-up is also outlined

	Baseline	Maintained	Developed	Follow-up	
	*n (%)*	*n (%)*	*n (%)*	*n (%)*	*p*
IBD-related symptoms	5 (7.6%)	0 (0%)	11 (16.7%)	11 (16.7%)	.210
IBS-like symptoms	6 (9.1%)	3 (4.5%)	34 (51.5%)	37 (56.1%)	<.001**

IBD Inflammatory Bowel Disease, *IBS* Irritable Bowel Syndrome

^*^p < .05. **p < .01

Regarding the second aim, 1.5% of the sample maintained at least one DSM diagnosis and 30.3% developed new DSM diagnoses. On the contrary, the percentage of IBD patients who maintained at least one DCPR syndrome was 54.5% whereas 21.2% developed new DCPR syndromes. The DCPR syndromes that were found to persist to some extent in IBD patients were allostatic overload (19.7% of the total sample), type A behavior (16.7%), alexithymia (7.6%), and demoralization (7.6%) (Table 2). In particular, the DCPR syndromes that persisted in more than half of the cases from baseline to follow-up were allostatic overload, Type A behavior and demoralization (Table 2).

## Discussion

The current study aimed to conduct an integrated assessment approach that encompasses the clinical and subclinical psychological profile of IBD outpatients in a longitudinal perspective, in order to provide a more comprehensive characterization of IBD patients.

Regarding psychiatric diagnoses, findings of the present study revealed a significant increase at follow-up, as only 4 (6.1%) patients had at least one DSM diagnosis at baseline, whereas 21 (31.8%) at follow-up. Among the most commonly observed diagnoses at follow-up, minor depression and generalized anxiety disorder were detected. Indeed, it was reported that anxiety and depressive disorders are prevalent [51] and can affect patients’ quality of life, as well as the course of IBD [52]. Moreover, in a review by Arp and colleagues [53] the prevalence of psychiatric comorbidities was high in patients with IBD and higher than in the background population. Further, a retrospective study by Marrie and colleagues [11] reported that individuals with Immune Mediated Inflammatory Disease (IMID), including IBD, have a non-specifically increased risk of psychiatric comorbidity with a cumulative incidence of depression of 20% five years after the IBD diagnosis.

About psychosomatic syndromes, the majority of the sample both at baseline and follow-up (71.2% and 75.5%, respectively) presented at least one DCPR syndrome. Among them, allostatic overload, alexithymia, type A behavior and demoralization were frequently observed both at baseline and follow-up in IBD patients, whereas persistent somatization was the only DCPR syndrome increased at follow-up. It is well known the relevance of the DCPR system in detecting psychological distress in medical disease settings, in comparison or integrated with the current psychiatric system. Indeed, DCPR syndromes were found to be transversally represented in most medical settings [39], even though in IBD patients had never been described. From the present study findings, it seems that more than a quarter of IBD patients present with allostatic overload, alexithymia or Type A behavior, both at baseline and follow-up. Patients with allostatic overload are thus characterized by feelings of being overwhelmed by the demands of daily life and may also present sleep disturbances, irritability, impaired social or occupational functioning [30]. Psychological stress has long been reported anecdotally to increase disease activity in IBD. Studies have confirmed that adverse life events and chronic stress increase the likelihood of relapse in patients with quiescent IBD [54]. IBD patients with alexithymia are inhibited concerning the experience and expression of emotions and show difficulties in communication [30]. Elevated levels of alexithymia were commonly observed in patients with IBD, especially in females [55], and this appeared to be associated with an increased likelihood of experiencing unfavorable clinical outcomes [19, 20]. IBD patients with Type A behavior are characterized by impatience and a sense of time-related pressure, irritability, and a competitive drive. The high frequency of Type A behavior in IBD patients and not only in cardiology settings, suggests that it might be considered as a relevant psychosomatic factor across a variety of clinical and preclinical conditions requiring a careful evaluation by clinicians [39]. Demoralized IBD patients present a feeling state characterized by their consciousness of having failed to meet their own expectations (or those of others) or being unable to cope with some pressing problems. Demoralized patients also experience prolonged and generalized feelings of helplessness. This phenomenon should not be ascribed to a normative reaction to stress or the presence of mood disorders in a subdued form [30]. In the literature, findings suggest a high prevalence of demoralization in the medically ill and the feasibility of a differentiation between demoralization and depression [56]. In the current study, the only diagnosis that had a significant increase at follow-up was persistent somatization. Indeed, 4 patients (6.1%) at baseline and 16 patients (24.2%) at follow-up presented this syndrome. Patients presenting persistent somatization complain about functional symptoms, such as IBS-like symptoms, causing distress, repeated medical care or impaired quality of life; they also exhibit additional symptoms of autonomic arousal also involving other organ systems and exaggerated side effects from medical therapy, indicating low sensations or pain thresholds and high suggestibility [30]. Consistently with the tendency of these patients to have difficulties expressing psychological distress, leading to its translation into physical symptoms, psychological comorbidity including somatization and perceived stress were found to be common in IBD [57, 58].

The comparison of well-being scores, as measured by the PWB-I at baseline and follow-up, did not show any significant results. However, in comparison with the PWB-I scores of a screening population for fecal occult blood test in a study conducted by Gostoli and colleagues [59], in the present investigation, the PWB-I scores of IBD patients without DCPR syndromes were similar to those of the screening population without DCPR syndromes, whereas the PWB-I scores of IBD patients with DCPR syndromes were slightly lower than those of the screening population with DCPR syndromes.

Concerning lifestyle-related behaviors, a notable and statistically significant increase in physical activity and the consumption of vegetables was detected. This could be the result of healthy lifestyle promoting prescriptions given by the gastroenterologist during the scheduled visits and followed by the patients, contrary to the literature contributions, which instead reported IBD patients’ difficulty in adhering to them [27]. It has been reported that physical activity represents a protective factor against IBD [22]. The literature also provides some guidelines on the optimal diet for these patients, suggesting that low-fiber diets may encourage intestinal inflammation, while high-fiber diets may provide protection against the inflammatory process [60]. In the present study, other healthy lifestyle behaviors were maintained, probably because most patients had been living with the disease for many years, before the first assessment, and thus they might have already adjusted some aspects of their habits. Consequently, at follow-up, the majority have continued to uphold these changes. This finding is in contrast with the study by Lo et al. [27], where IBD outpatients were found to not practice such recommendations. As said before, it is likely that the gastroenterologists involved in the present study posed a great attention on healthy lifestyle.

Concerning the IBD related symptoms and IBS-like symptoms, whilst nearly all the patients were asymptomatic at baseline, only IBS-like symptoms such as abdominal pain, bloating, diarrhea and watery stools showed a significant increase after 4-year follow-up. On the same line, contributions in literature showed a higher prevalence of IBS-like symptoms in patients with IBD than in the general population [61]. Such symptoms were associated with psychological comorbidity, such as anxiety and depression [62]. According to Spiller and Major [63], a dysregulated interaction between peripheral factors (i.e., microbiota and post-inflammatory changes) and central factors (stress, psychological comorbidity), might generate such gastrointestinal symptoms. Furthermore, in the present study, findings of increased persistent somatization at follow-up might result in a higher burden of IBS-like symptoms.

Regarding the second aim, it was observed that only 1.5% of the IBD patients maintained at least one DSM diagnosis, despite in the literature Bisgaard and colleagues [28] found that patients with IBD had higher risk of anxiety and depression continuing until at least ten years after IBD diagnosis. On the other hand, our findings showed that more than half of the patients maintained psychosomatic syndromes between baseline and follow-up. The most stable DCPR syndromes included allostatic overload, type A behavior and demoralization. To the best of our knowledge, there is a lack of literature specifically addressing this topic but there are some notable contributions in other medical settings. For example, patients with post-coronary artery bypass grafting and those with Implantable Cardioverter Defibrillators (ICD) showed sustained DCPR syndromes over time. The 1-month and 6/8-year post-surgery assessments for bypass patients confirmed stability in type A behavior, irritable mood, demoralization and persistent somatization [64]. The latter syndromes persisted over a year in ICD patients [65]. In line with our findings, DCPR categories remained stable in individuals over time in both cases. The stability of DCPR syndromes might as well have resulted in increased burden of IBS-like symptoms along the follow-up period, as previously described. Moreover, unlike depression, demoralization was found to rarely persist as an uninterrupted condition for any substantial duration, in different medical settings (e.g., gastroenterology, cardiology, endocrinology, and oncology) [66]. However, this is in contrast with the findings of the present study, where more than half of the patients affected by demoralization at baseline presented with persistent demoralization at follow-up.

The current study shows some limitations such as small sample size, absence of a control group, distinction between IBD- and IBS-like symptoms not always possible and data collection method, which changed between the initial and follow-up assessments. Indeed, during the baseline assessment, interviews were performed face-to-face, while follow-up assessments were conducted over the phone. This variation in data collection methods might have introduced factors such as participants’ fatigue, especially given the overall length of the interviews, potentially leading to less attentive responses. In addition, the telephone-based approach and the absence of in-person contact could have influenced the relationship between the interviewer and the patients, especially with older participants.

Despite the mentioned limitations, the strengths of this study include the presence of a comprehensive assessment of clinical and sub-clinical aspects of IBD patients, the longitudinal design of the study and the use of measure according to clinimetric validity.

## Conclusion

These longitudinal study findings revealed clinical and persistent subclinical distress in a sample of IBD outpatients longitudinally assessed. It seems that, whereas psychiatric conditions are more changeable over time, psychosomatic syndromes such as allostatic overload, type A behavior, alexithymia and demoralization represent persistent unhealthy attitudes in IBD patients at a long-term follow. These findings, together with the development of persistent somatization at follow-up, might have an impact on the increase of IBS-like symptoms, whereas after 4-year follow-up IBD symptoms were managed. Since it has been advocated the need for a comprehensive assessment and management of IBD, in order to develop more targeted treatment, an integrated assessment through the DCPR system enables the identification of subclinical aspects not detected by traditional tools, as vulnerability factors of the disease. From a secondary prevention perspective, studying the persistence over time of certain DCPR syndromes in patients with IBD could be useful to monitor the psychophysical conditions of IBD patients.

## Data Availability

The data that support the findings of this study are available on request from the corresponding author. The data are not publicly available due to privacy or ethical restrictions.
